# Comparing psychometric characteristics of a computerized cognitive test (BrainCheck Assess) against the Montreal cognitive assessment

**DOI:** 10.3389/fpsyg.2024.1428560

**Published:** 2024-09-03

**Authors:** Duong Huynh, Kevin Sun, Reza Hosseini Ghomi, Bin Huang

**Affiliations:** ^1^BrainCheck Inc., Austin, TX, United States; ^2^Frontier Psychiatry, PLLC, Billings, MT, United States

**Keywords:** dementia, mild cognition impairment, BrainCheck, MOCA, neuropsychology, Alzheimer, cognition, computerized cognitive assessment

## Abstract

**Introduction:**

Previous validation studies demonstrated that BrainCheck Assess (BC-Assess), a computerized cognitive test battery, can reliably and sensitively distinguish individuals with different levels of cognitive impairment (i.e., normal cognition (NC), mild cognitive impairment (MCI), and dementia). Compared with other traditional paper-based cognitive screening instruments commonly used in clinical practice, the Montreal Cognitive Assessment (MoCA) is generally accepted to be among the most comprehensive and robust screening tools, with high sensitivity/specificity in distinguishing MCI from NC and dementia. In this study, we examined: (1) the linear relationship between BC-Assess and MoCA and their equivalent cut-off scores, and (2) the extent to which they agree on their impressions of an individual’s cognitive status.

**Methods:**

A subset of participants (*N* = 55; age range 54–94, mean/SD = 80/9.5) from two previous studies who took both the MoCA and BC-Assess were included in this analysis. Linear regression was used to calculate equivalent cut-off scores for BC-Assess based on those originally recommended for the MoCA to differentiate MCI from NC (cut-off = 26), and dementia from MCI (cut-off = 19). Impression agreement between the two instruments were measured through overall agreement (OA), positive percent agreement (PPA), and negative percent agreement (NPA).

**Results:**

A high Pearson correlation coefficient of 0.77 (CI = 0.63–0.86) was observed between the two scores. According to this relationship, MoCA cutoffs of 26 and 19 correspond to BC-Assess scores of 89.6 and 68.5, respectively. These scores are highly consistent with the currently recommended BC-Assess cutoffs (i.e., 85 and 70). The two instruments also show a high degree of agreement in their impressions based on their recommended cut-offs: (i) OA = 70.9%, PPA = 70.4%, NPA = 71.4% for differentiating dementia from MCI/NC; (ii) OA = 83.6%, PPA = 84.1%, NPA = 81.8% for differentiating dementia/MCI from NC.

**Discussion:**

This study provides further validation of BC-Assess in a sample of older adults by showing its high correlation and agreement in impression with the widely used MoCA.

## Introduction

Millions of older adults around the world suffer from Alzheimer’s Disease and AD-Related Dementias (AD/ADRD) yet it is still underdiagnosed and often detected in its late stages (Lang et al., 2017; Amjad et al., 2018; Liu et al., 2023), particularly in primary care settings (Borson et al., 2006). The implications of an early diagnosis can prompt the delivery of timely interventions, open access to resources limited by medical necessity, and incite planning ahead for future support, living, and safety of the person living with dementia. This has been further accentuated in light of the United States Food and Drug Administration’s approval of two novel treatment options of Alzheimer’s Disease [Lecanemab (Van Dyck et al., 2023) and Donanemab (Reardon, 2023)]. The earlier the diagnosis, the more likely treatments and interventions will be able to slow disease progression (Rasmussen and Langerman, 2019) which can aid in longer preservation of function.

In clinical practice, a common standard has been to utilize paper based instruments such as the Montreal Cognitive Assessment (MoCA) or the Mini-Mental State Examination (MMSE) to measure cognition for the screening of AD/ADRD. Of these, the MoCA is generally accepted to be among the most comprehensive and robust screening tools, with high sensitivity/specificity in detecting cognitive impairment (Nasreddine et al., 2005; Carson et al., 2018; Dautzenberg et al., 2020). It covers a broad range of domains essential for cognitive assessment and has been well-validated in many studies (Nasreddine et al., 2005; Smith et al., 2007; Luis et al., 2009; Roalf et al., 2013). However, these paper-based instruments have certain limitations. Firstly, they lack precision needed for measuring response time, a significant aspect when measuring cognitive processes. Secondly, these tests are time-and labor-intensive as they require verbal administration and manual scoring by a trained or licensed administrator. Moreover, these administration and scoring processes open up opportunities of inconsistency and subjectivity which makes it susceptible to interrater variability (Price et al., 2011; Hilgeman et al., 2019; Cumming et al., 2020). Lastly, although these tools have been available for many years, the detection rate of cognitive impairment and AD/ADRD has not been significant (Chodosh et al., 2004; Lang et al., 2017), leading to the conclusion that additional tools are necessary to improve the rate and the timeliness of diagnosis. Doing so would naturally lower the high burden of care that comes with late stage clinical symptoms driving significant costs in our healthcare system (Alzheimers Association, 2019; Matthews et al., 2019).

Computerized cognitive assessments inherently offer greater efficiency and feasibility, thanks to their self-administration capability, remote accessibility, automated scoring, and ability to seamlessly integrate with electronic health records (EHR). Designed to overcome limitations in traditional instruments and align with clinical workflow, BrainCheck has developed a digital cognitive assessment tool, BrainCheck Assess (BC-Assess), to objectively measure multiple cognitive domains to aid early detection of AD/ADRD. BC-Assess takes 10–15 min to complete and can be self-administered remotely or administered in person by clinical staff with minimal training required. A comprehensive clinical report is immediately generated with test results and applicable knowledge to aid in clinical decision making. Multiple validation studies demonstrated that BC-Assess could reliably and sensitively identify those suffering from concussion (Yang et al., 2017) or age-related cognitive impairment (Groppell et al., 2019; Ye et al., 2022). In the latest study (Ye et al., 2022) with 99 participants, we found BC-Assess overall scores were significantly different across the three groups [normal cognition (NC), mild cognitive impairment (MCI), and dementia (DEM)]. Results showed ≥88% sensitivity and specificity for separating DEM and NC, and ≥77% sensitivity and specificity in differentiating MCI with NC and DEM. However, we have not provided a crosswalk between BC-Assess and a traditional paper-based cognitive test that allows for converting scores from one test to the other and supports interpretations of its results.

The aim of this study was to validate BC-Assess against the MoCA through comparison of their scores. In this study, we retrospectively analyzed clinical data collected from two previous studies (Groppell et al., 2019; Ye et al., 2022) to examine: the linear relationship between BC-Assess overall score and MoCA total score and their equivalent cut-off scores, and the extent to which they agree on their impressions of an individual’s cognitive status.

## Materials and methods

### Participants

A subset of 55 participants (age range 54–94, mean/SD = 79.9/9.5; 70.9% Female) from two previous studies (Groppell et al., 2019: *N* = 35; Ye et al., 2022: *N* = 20) who completed both the MoCA and BC-Assess were included in this analysis. Table 1 summarizes demographic characteristics of the participants from each study. The 35 participants from the Groppel et al. study were recruited from two assisted-living facilities in Houston, TX. The 20 participants from the Ye et al. study were recruited from a research registry maintained by the University of Washington (UW) Alzheimer’s Disease Research Center Alzheimer’s Disease Research Center associated with UW Medicine’s Memory and Brain Wellness Center.

**Table 1:** Demographics and summary statistics of MoCA and BC-Assess scores of the study sample.

Demographic characteristics	Groppell et al. (2019) (*n* = 35)	Ye et al. (2022) (*n* = 20)	Total (*N* = 55)
Gender, No. (%)			
Female	31 (88.6%)	8 (40%)	39 (70.9%)
Male	4 (11.4%)	12 (60%)	16 (29.1%)
Age, years			
Mean (SD)	85.1 (6.4)	70.9 (7.2)	79.9 (9.5)
Range	63–94	54–80	54–94
Administration type, No. (%)			
On-site BC-Assess	35 (100%)	10 (50%)	45 (81.8%)
Remote BC-Assess	0 (0%)	10 (50%)	10 (18.2%)
Device, No. (%)			
BC-Assess on iPad	35 (100%)	15 (75%)	50 (90.9%)
BC-Assess on laptop/desktop	0 (0%)	5 (25%)	5 (9.1%)
MoCA total score			
Mean (SD)	14.9 (8.1)	23.4 (4.2)	18.0 (8.0)
Range	0–28	17–29	0–29
BC-Assess standardized overall score			
Mean (SD)	55.7 (30.5)	82.7 (23.9)	65.5 (31.1)
Range	0–96.6	44.4–128.3	0–128.3

### Data collection

Participants in the Groppel et al. group completed both the MoCA and BC-Assess on the same day at the testing centers. In the Ye et al. group, the time interval between the two tests and administration type varied among participants. For this group, except for one participant completing the MoCA 28 days after the BC-Assess, the remaining 19 participants completed the BC-Assess 39–299 days after the MoCA. MoCA scores for these participants were obtained from EHR where only the total scores were available. Of these 19 participants, 10 were administered the BC-Assess battery remotely over a video call with the moderator due to the COVID-19 pandemic.

### MoCA total scores

The MoCA is a cognitive screening test that measures multiple cognitive domains: short-term memory recall, visuospatial abilities, executive functioning, phonemic fluency, verbal abstraction, attention, concentration, language, and time and place orientation. The total possible score is 30 points. Cut-off scores of 19 and 26 are typically used to differentiate dementia from MCI and MCI from NC, respectively (Davis et al., 2013).

### BC-Assess standardized overall scores

The BC-Assess consisted of six individual assessments: Immediate/Delayed Recognition, Digit Symbol Substitution, Stroop, Trails Making A/B. A detailed description of these assessments can be found in our previous study (Yang et al., 2017). Performance on each assessment was quantified by either accuracy- or reaction time-based measures (Table 2). The BC-Assess raw overall score was calculated as the mean of performance scores from all assessments in the battery (except the Trails Making B), where each assessment-specific score had been transformed from its natural range into a common range [0,100] using the formula in Table 2. An overall z-score was then calculated using population mean and standard deviation values from the BrainCheck normative database to correct for age and testing device differences across participants. The BC-Assess standardized overall score (BC-SS) was the *z*-score rescaled to describe each individual’s overall score relative to a population mean of 100 and a population standard deviation of 15. The BC-SS ranges from 0 to 200. This score was clipped if it fell out of the range.

**Table 2:** Raw score (RS) metric and transformed score (TS) calculation for each assessment.

Assessment	Raw score (RS) metric	Transformed score (TS)
Immediate/delayed recognition	Number of correct responses	TS = 100*RS/MAX^1^
Trails making A	Median reaction time	TS = 100*(1-RS/MAX)
Stroop	Median reaction time	TS = 100*(1-RS/MAX)
Digit symbol substitution	Number of correct responses per second	TS = 100*RS/MAX

^1^MAX represents the population maximum score of the assessment across all individuals in the BrainCheck normative database.

### Data analysis

This study analyzed the relationship between BC-SS and MoCA total scores. Linear regression was used to find the linear relationship between the two test scores. The best fit model was used to calculate equivalent cut-off scores for BC-SS based on those originally recommended for the MoCA to differentiate MCI from NC (cut-off = 26), and dementia from MCI (cut-off = 19). Impression agreement between the two instruments were measured through overall agreement (OA), positive percent agreement (PPA), and negative percent agreement (NPA).

The above analysis assumed that the cognitive status of participants had not changed significantly between the two tests, which might not be true for participants from the Ye et al. study. The effect of remote testing was also assumed to be negligible for those completing the BC-Assess remotely. For comparison purposes, the same analysis was performed on the entire study sample and on only the Groppel et al. group who took the BC-Assess and MoCA in-person and on the same day.

## Results

**Figure 1:**
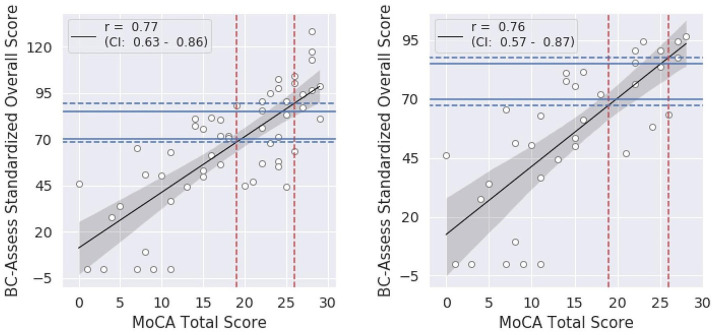
Relationship between the BC-SS and MoCA total score for the entire sample **(left)** and for only the Groppel et al. group **(right)**: black lines = linear regression models, shaded areas = 95% confidence intervals. Blue dashed lines represent BC-SS calculated from the model for each MoCA cut-off value (red dashed lines). Blue solid lines show BC-SS cut-offs currently recommended.

Summary statistics of the BC-SS and MoCA total score across participants in each group and the entire sample are shown in Table 1. A high correlation between the two test scores was observed for both the entire study sample (Pearson correlation coefficient *r* = 0.77; 95% Confidence Interval = 0.63–0.86; Figure 1 - left panel) and for only the Groppel et al. group (*r* = 0.76; 95% Confidence Interval = 0.57–0.87; Figure 1 - right panel). Coefficient estimates and goodness of fit of the linear regression model characterizing the relationship between the two scores are shown in Table 3 for each case.

**Table 3:** Linear regression model fitting (BC-SS = *Intercept* + *Slope* x MoCA Total Score) for the entire sample and for only the Groppel et al. group.

Coefficients	Entire sample			Groppel et al.		
	Estimate	*p*-value	Adjusted *R*-squared	Estimate	*p*-value	Adjusted *R*-squared
Intercept	11.25	0.102	0.585	12.44	0.099	0.564
Slope	3.01	<0.001		2.90	<0.001	

According to the linear relationship found for the entire sample, MoCA cutoffs of 26 and 19 correspond to BC-Assess scores of 89.6 and 68.5, respectively. Similar BC-Assess scores, 87.8 and 67.5, respectively, were obtained for the case where only the Groppel et al. group was included. Results based on either set of participants are close with the currently recommended BC-Assess cutoffs (i.e., 85 and 70).

Tables 4, 5 show the confusion matrices for comparisons of the two tests’ impressions according to their recommended cut-offs for the entire study sample and for only the Groppel et al. group, respectively. For both sets of participants, a high degree of agreement in their impressions were observed:

- Entire sample: OA = 70.9%, PPA = 70.4%, NPA = 71.4% for differentiating dementia from MCI/NC; and OA = 83.6%, PPA = 84.1%, NPA = 81.8% for differentiating dementia/MCI from NC.
- Groppel et al. group: OA = 77.1%, PPA = 78.3%, NPA = 75.0% for differentiating dementia from MCI/NC; and OA = 85.7%, PPA = 87.1%, NPA = 75.0% for differentiating dementia/MCI from NC.

**Table 4:** Number of participants from the entire study sample classified as NC, MCI, and Dementia by BC-Assess (rows) and MoCA (columns) based on their recommended cut-offs.

BC-Assess	MoCA			
	Dementia	MCI	NC	Total
Dementia	19	7	1	27
MCI	8	3	1	12
NC	0	7	9	16
Total	27	17	11	55

**Table 5:** Number of participants from the Groppel et al. group classified as NC, MCI, and Dementia by BC-Assess (rows) and MoCA (columns) based on their recommended cut-offs.

BC-Assess	MoCA			
	Dementia	MCI	NC	Total
Dementia	18	2	1	21
MCI	5	2	0	7
NC	0	4	3	7
Total	23	8	4	35

## Discussions

This retrospective study sought to compare the psychometric characteristics of a digital cognitive assessment (BC-Assess) against the widely used paper MoCA based on existing data collected from a cohort of 55 participants in two previous studies. We found a strong linear association between scores from these two tests and a high agreement in their impression of an individual’s cognitive status. The MoCA cut-off scores commonly used for detecting dementia (cut-off = 19) and MCI (cut-off = 26) corresponded to BC-Assess scores of 68.5 and 89.6, respectively, which align well with the cut-off values recommended by BrainCheck (70 and 85). These results further demonstrate the validity of BC-Assess as a cognitive screening tool.

Over the years, BC-Assess has gained good adoption among primary care providers for its demonstrated high diagnostic performance (Groppell et al., 2019; Ye et al., 2022), high usability and feasibility of implementation (BrainCheck, 2019, 2020), and its likeliness to secure medical claim reimbursement. However, many providers who have been trained to use the MoCA are not familiar with the clinical meaning of BC-Assess scores and how they are related to the MoCA scores they have been trained on. This limits these providers’ usage of BC-Assess with their patients and may hinder the widespread screening of cognitive impairment in the community. By directly comparing BC-Assess against the MoCA and providing BC-Assess equivalent cut-off scores for detecting MCI and dementia, this study offers additional support to providers in interpreting BC-Assess results and in decision making, which is expected to facilitate their use of this computerized tool in clinical practice.

To promote widespread detection of cognitive impairment and AD/ADRD, it is critical to equip primary care providers with assessment tools that are not only validated and reliable but also easy to administer and seamlessly integrated into their routine workflows (Mattke et al., 2023). In the primary care setting, the limited time of patient visits, which typically last an average of 18.9 min (Neprash et al., 2023), and the fact that geriatric patients tend to have multiple health conditions that rank over cognitive concerns, introduce the major challenge for deploying cognitive assessments. As a computerized cognitive assessment, BC-Assess holds promise for meeting these criteria as demonstrated in multiple case studies (BrainCheck, 2019, 2020). To gain a better understanding of BC-Assess’ usability and feasibility of implementation in comparison with the MoCA, future studies with comprehensive evaluations of the deployment process for each tool across a wide range of real-world settings are needed.

The results in this study should be interpreted within the context of some limitations. Firstly, the BC-Assess and MoCA were taken at different time points for a large proportion of participants. For these participants, the time interval between the two tests was up to several months. A participant’s cognitive status might change substantially during this time due to normal aging and other medical issues, which is especially true for older adults. This could lead to unsound results because analysis of the linear relationship between scores from the two tests assumed that there was no or minimal intra-individual variability in cognition across the two measures. Another factor that could have contaminated the results is the inconsistency of administration type across participants. With 10 out of 55 participants completing the BC-Assess remotely, the effects of self-administration and variability of testing environment on test scores, if any, would need to be taken into account. Secondly, the study sample lacked information about education level and was relatively small and homogeneous in regard to age and range of cognitive performance, which may impact the generalizability of the findings. More than 90% of participants were over 65 years of age and nearly 60% of which were older than 80. Similarly, roughly 50% of participants scored lower than 19 in the MoCA test. Only 30% and 20% of participants scored between 19 and 26 and higher than 26, respectively. A larger sample size and more symmetric distributions of education level, age, and range of cognitive performance, are imperative to acquire a more precise and generalized relationship between the two tests. In addition, this study also lacked a thorough comparison between the BC-Assess and MoCA by cognitive domain due to missing information of MoCA subscales for participants from the Ye et al. study. Future studies utilizing BC-Assess and the MoCA or other cognitive measurements should include more controlled procedures to further corroborate these results.

## Data Availability

The datasets presented in this article will be made available upon reasonable request to the corresponding author.
